# Capillary Flow Profile Analysis on Paper-Based Microfluidic Chips for Classifying Astringency Intensity

**DOI:** 10.3390/s25165068

**Published:** 2025-08-14

**Authors:** Daesik Son, Junseung Bae, Chanwoo Park, Jihoon Song, Soo Chung

**Affiliations:** 1 Department of Biosystems Engineering, Seoul National University, Seoul 08826, Republic of Korea; ftiu7957@snu.ac.kr (D.S.); bjs037@snu.ac.kr (J.B.); successful538@snu.ac.kr (C.P.); wingspec@snu.ac.kr (J.S.); 2 Research Institute of Agriculture and Life Sciences, Seoul National University, Seoul 08826, Republic of Korea; 3 Integrated Major in Global Smart Farm, Seoul National University, Seoul 08826, Republic of Korea

**Keywords:** astringency intensity, microfluidic paper-based analytical device, capillary profile, machine learning

## Abstract

Astringency, a complex oral sensation resulting from interactions between mucin and polyphenols, remains difficult to quantify in portable field settings. Therefore, quantifying the aggregation through interactions can enable the classification of the astringency intensity, and assessing the capillary action driven by the surface tension offers an effective approach for this purpose. This study successfully replicates tannic acid (TA)–mucin aggregation on a paper-based microfluidic chip and utilizes machine learning (ML) to analyze the resulting capillary flow dynamics. Aggregates formed by mixing mucin with TA solutions at three concentrations showed that higher TA levels led to greater aggregation, consequently reducing the capillary flow rates. The flow dynamics were consistently recorded using a smartphone mounted within a custom 3D-printed frame equipped with a motorized sample loading system, ensuring standardized experimental conditions. Among eight trained ML models, the support vector machine (SVM) demonstrated the highest classification accuracy at 95.2% in distinguishing the astringency intensity levels. Furthermore, fitting the flow data to a theoretical capillary flow equation allowed for the extraction of a single coefficient as an input feature, which achieved comparable classification performance, validating the simplified feature extraction strategy. This method was also feasible even with only a portion of the initial data. This approach is simple and cost-effective and can potentially be developed into a portable system, making it useful for field analysis of various liquid samples.

## 1. Introduction

Astringency is defined as a sensory experience of dryness, roughness, and tightening in the oral cavity, commonly observed after the consumption of foods and beverages such as unripe fruits, berries, wine, and tea [1]. Astringency is primarily caused by polyphenols such as tannins. Despite the health benefits of these compounds, excessive astringency can lead to an unpleasant oral sensation, and it is therefore considered an important factor in food quality that significantly affects the consumer’s sensory experience [1,2]. However, individual perception of astringency is inherently subjective and may differ due to physiological and genetic variations [3]. Therefore, developing an objective method for evaluating the intensity of astringency in foods is important, as it could affect consumers’ decision-making when selecting products.

The current methods for assessing astringency primarily involve aggregation-based techniques centered on hydrophobic interactions between polyphenols and proteins. One approach utilizes the increase in the turbidity caused by the formation of tannic acid (TA)–mucin aggregates, which scatter light within the solution [4]. Another method quantifies the amount of tannins by measuring differences in the absorbance resulting from the aggregation of tannins with methylcellulose [5]. However, these methods have inherent limitations: they are susceptible to interference from the ambient lighting conditions during measurement, their accuracy can be compromised by the intrinsic color of the sample, and they require expensive equipment. Recently, electrochemical approaches have been proposed, including the use of ion-selective electrodes [6], hydrogel sensors mimicking the human salivary layer based on a TA–mucin aggregation sensor [7], and electrochemical biosensors utilizing the adsorption of salivary proteins onto gold screen-printed electrodes [8]. These electrochemical methods offer advantages such as a wide detection range and high sensitivity. However, they also present limitations, including the need for expensive electrodes and analytical equipment, as well as the complexity of the fabrication processes.

Paper-based microfluidic chips are structurally simple, enabling miniaturization, faster analysis, and spontaneous liquid flow through capillary action without the need for external or internal pumps [9]. Therefore, methods using these are well-suited for on-site applications and have been widely used to detect various substances in fields such as healthcare [10], food analysis [11], and environmental analysis [12]. Detection methods using paper-based microfluidic chips typically rely on two main mechanisms: (i) monitoring the distance traveled by the fluids, which move through the microspaces between the cellulose fibers via capillary action and interact with the fibers [13], or (ii) observing color changes resulting from interactions between immobilized reagents on the fibers and the fluids [10,14]. In recent studies, colorimetric approaches have been employed on paper-based microfluidic chips to detect taste-related compounds, including glutamate—associated with the umami taste—and polyphenols—responsible for astringency [11,15]. In addition, simultaneous detection of sugars such as sucrose, fructose, and glucose has also been demonstrated using similar platforms [16]. However, colorimetric detection methods face limitations when applied to liquid food samples due to interference from the inherent coloration of the samples.

Recently, flow dynamics-based methods utilizing capillary action in paper-based microfluidic chips have been proposed [13]. Based on this approach, machine learning (ML) has been applied to analyze the flow profiles of the moving fluids, leading to the successful classification of bacterial species [17] and types of oils [18] with high accuracy. In a similar approach, perfluorinated carbon alkyl chains (PFOA), a harmful substance, were detected by analyzing changes in the velocity profiles resulting from alterations in the surface tension caused by their interaction with bovine serum albumin (BSA) [19]. These studies are based on analyzing variations in the flow profiles that arise from changes in the interaction capabilities of the flowing liquid and the cellulose fibers in paper-based microfluidic chips, governed by molecular properties such as the charge, polarity, and hydrophobicity [18].

Mucin is a high-molecular-weight glycoprotein characterized by a peptide backbone with numerous glycan side chains attached in a bottle-brush structure, resulting in a high hydration capacity and high viscosity [20]. Tannic acid is a polyphenol containing multiple phenolic hydroxyl groups (-OH), known to interact with various biomacromolecules and contribute to increased viscosity [21], particularly by forming aggregates through interactions with mucin. The aggregation and subsequent precipitation of these complexes within the oral cavity is responsible for the perception of astringency [2]. Therefore, depending on the concentration of TA—and consequently the intensity of astringency—the capabilities of the mucin–TA mixture to interact with the paper fibers would vary. Based on this understanding, a simple system composed of paper and a camera could be utilized to classify the intensity of astringency in a field-friendly and low-cost manner.

In this study, we aimed to classify the intensity of astringency by analyzing flow profile variations in paper-based microfluidic chips. TA, a major compound responsible for inducing astringency, was mixed with mucin solutions at three concentrations within the human sensory detection range, and the resulting flow profiles on the paper-based microfluidic chips were analyzed using ML models. A controlled flow profile acquisition system was utilized using a motor-driven motion controller and a frame fabricated with a 3D printer to ensure a consistent flow profile. This system can be further miniaturized into a simple, portable platform, making it a promising method for on-site classification of the astringency intensity.

## 2. Materials and Methods

### 2.1. Sample Preparation

TA powder (tannic acid ACS reagent, Sigma-Aldrich, St. Louis, MO, USA) was dissolved in a pH 3 buffer solution (SAMCHUN, Pyeongtaek, Republic of Korea) to prepare concentrations of 0.2 g/L, 1.0 g/L, and 2.0 g/L. Mucin powder (Type III, from porcine stomachs, Sigma-Aldrich, St. Louis, MO, USA) was dissolved in a pH 3 buffer solution to prepare a 4 g/L mucin solution. For sufficient dissolution, a hotplate stirrer (Hotplate Stirrer Digital Control, 260 × 260 MSH-30D, DAIHAN Scientific, Seoul, Republic of Korea) was used to stir the mucin solution at 400 rpm for 2 h and the TA solution for 5 min at room temperature. Subsequently, 100 mL of each of the TA solutions at three different concentrations was mixed with the mucin solution at a 1:1 volume ratio and stirred for an additional 20 min to facilitate the formation of mucin–TA aggregates. After mixing, 3.15 mL of the resulting solution was transferred into a cuvette (HRA-2712120, RATIOLAB, Dreieich, Germany) for use in measuring the fluid profiles on paper-based microfluidic chips, as described in Section 2.3.

### 2.2. Paper-Based Microfluidic Chips and Equipment for Flow Velocity Profile Measurement

#### 2.2.1. Paper-Based Microfluidic Chips

**Figure 1:**
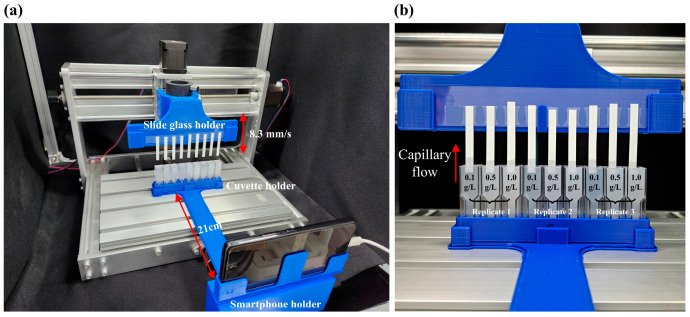
(**a**) Motor-driven motion-controlled sample loading and video recording system; (**b**) 3D-printed holder and 9 microfluidic channels attached to two slide glasses for three different concentrations of tannic acid with three replicates each. Each cuvette and paper-based microfluidic chip and the smartphone were securely fixed in place using a 3D-printed holder to ensure consistent data acquisition. Although the lengths of the paper-based microfluidic chips exhibited slight variations, the lowest points at which the samples were loaded were aligned on the same horizontal plane.

Paper-based microfluidic chips were created using cellulose chromatography paper (Whatman 1 CHR, Whatman, Maidstone, UK), which has been used in a previous study [18], cut to 5 mm in width using a utility knife and a ruler to create a narrow channel for microfluidic flow. The paper used in this experiment was originally square-shaped and was cut without considering the fiber orientation. To minimize the bending of the paper-based microfluidic chip and ensure that the bottom edges of all the chips were aligned on the same horizontal plane, a quarter of one end was fixed to a slide glass using adhesive tape, as shown in Figure 1b. The distance from the bottom edge of the slide glass to the end of the paper-based microfluidic chip was 29.2 mm. The flow profile was acquired only before reaching the bottom edge of the slide glass.

#### 2.2.2. Motor-Controlled Sample Loading System

To position one side of the paper-based microfluidic chip in the cuvette with the sample at a uniform speed and depth, a motor-driven motion platform capable of precise vertical movement was utilized, based on a stepping motor (US-17HS4401S, Usongshine, Shenzheng, China). G-code parsing and streaming were performed using two open-source Python libraries, grbl-streamer (v2.0.2) [22] and gcode-machine (v1.0.3) [23]. Using the gcode-machine library, the motion was controlled with a resolution of 0.01 mm and a minimum velocity of 0.03 mm/s. In this study, the paper was inserted into the sample at a deliberately slow speed of 8.3 mm/s.

#### 2.2.3. Devices and Conditions for Velocity Monitoring

To enable the loading of the sample onto the motor-driven motion platform, a holder allowing for the insertion and removal of a slide glass attached to the paper-based microfluidic chip was designed using Fusion 360 (Autodesk, San Francisco, CA, USA) and fabricated with a 3D printer (3DWOX7X, Sindoh, Seoul, Republic of Korea) and was integrated with the platform as shown in Figure 1a. Additionally, a holder was fabricated using a 3D printer to maintain a constant distance between the smartphone and the cuvette containing the sample for fluid flow imaging. The holder was designed with a horizontally fixed shaft of 21 cm in length, onto which the smartphone holder and cuvette holder were separately attached. During imaging, the smartphone was fixed vertically relative to the ground, and nine cuvettes containing TA–mucin mixtures were positioned directly beneath each paper-based microfluidic chip using the cuvette holder. Velocity profile videos were captured using a smartphone (GALAXY A53, SAMSUNG, Suwon, Republic of Korea) configured at 2× optical magnification, a 1/50 shutter speed, ISO 125 [24], and 30 fps. Each recording was conducted for 61 s at a resolution of 1920 × 1080 pixels.

### 2.3. Velocity Profile Measurement

Using the flow profile measurement system designed as shown in Figure 1a, three cuvettes containing TA solutions at each of three different concentrations (0.1, 0.5, and 1.0 g/L), mixed sufficiently with mucin solutions, were prepared for each concentration, resulting in a total of nine cuvettes. These nine cuvettes were prepared to allow for three repeated measurements within a single experimental session. The motor-driven motion system was controlled at a deliberately slow speed of 20 mm/s to minimize the influence of the sample loading methods, such as surface disturbances caused by rapid motion, on the fluid flow behavior [9]. In addition, the motor movement was precisely adjusted to submerge each of the nine papers 3 mm below the sample surface.

Upon contact with the sample surface in the cuvette, the paper induced an upward fluid flow driven by capillary action. These fluid profiles were recorded using a smartphone camera. A total of 14 measurements were conducted, with each measurement comprising nine samples at three different concentrations of TA. Consequently, 42 profiles were obtained for each concentration, resulting in a total of 126 profiles.

### 2.4. Data Frame and Preprocessing

#### 2.4.1. Raw Flow Profile Dataset

From the recorded videos, images were extracted at 30 frames per second, and the pixel value changes in the wet regions were analyzed. Using a custom Python (version 3.13.3) script, the highest point reached by the fluid in each frame was determined. To address potential errors caused by the initial instantaneous contact between the paper and the fluid surface, the pixel coordinates from the first 30 frames (1 s) after contact were used as a reference. The difference between these initial coordinates and the highest-point coordinates in subsequent frames was calculated and defined as the fluid height. Since the wetted region could not decrease in size during the progression of capillary flow, if a lower fluid height was detected compared to that in the previous frame, the previous value was preserved to ensure a monotonic increase.

To minimize errors caused by positional differences and the random fiber structure of the paper-based microfluidic chips during the simultaneous measurement of three different tannic acid concentrations in a single session, the average of the three simultaneously acquired datasets for each concentration was used. The acquired dataset consisted of two-dimensional time-series data, which recorded the changes in the fluid height over time for each sample. The *x*-axis represented the frame index corresponding to the progression of time, while the *y*-axis indicated the fluid height at each time point, defined as the coordinate difference in the same-resolution images.

For each frame, the fluid height values were normalized using min–max scaling, resulting in all the values being rescaled to within a range between 0 and 1. In this study, the collection of time-series data representing the time-dependent change in the fluid height for all 126 samples was defined as the “raw dataset”. Additionally, the averaged data from the three replicates for each tannic acid concentration obtained in a single measurement session were defined as the “mean dataset,” reducing the total number of profiles to 42.

#### 2.4.2. Model-Fitted Flow Profile Dataset

In capillary-driven flow, the relationship between the time (*t*) and the fluid travel distance (Ɩ) is described by Equation (1), governed by the capillary radius (*R*), the interfacial tension at the wetting front ($\gamma_{LG}$), the contact angle (*θ*), and the dynamic viscosity of the liquid (*μ*) [9]. This relationship can be simplified, as shown in Equation (2), into a basic form where the distance traveled is proportional to the square root of the time. As explained in Section 2.4.1, the exclusion of the initial 1 s (30 frames) inherently determined the horizontal shift, and thus only the constant b was added in Equation (3) to account for the vertical translation of the function.

(1) $$\frac{{Ɩ}^{2}}{t}\propto \;\frac{R\gamma_{LG}cos\theta }{2\mu }$$

(2) $$Ɩ=a\sqrt{t}$$

(3) $$Ɩ=a\sqrt{t+1}+b$$

The mean dataset, defined in Section 2.4.1, was fitted to Equation (3) by applying the curve fit function from the Python library, using the average displacement over time. From this fitting, the constant *a* in the equation was calculated for each sample. The resulting constants were organized into a separate dataset, referred to as the “fit coefficient dataset”. While the raw dataset and mean dataset each contained 1800 frame-based features, the fit coefficient dataset consisted of only a single feature per sample, corresponding to the coefficient *a* from Equation (3).

### 2.5. Machine Learning Models and Hyperparameter Tuning

Eight machine learning (ML) models (support vector machine (SVM), Linear Regression (LR), multi-layer perceptron (MLP), k-nearest neighbors (kNNs), Random Forest (RF), Linear Discriminant Analysis (LDA), Naïve Bayes (NB), Decision Tree (DT)) were trained to select the optimal classification model for four datasets (raw, mean, fit coefficient, and fit coefficient over 15 s). All the ML models were implemented using the sci-kit learn library [25] in Python.

To optimize the hyperparameters for each machine learning (ML) model, the accuracies of the eight ML models were compared using a 3-fold cross-validation (CV) for each dataset. The optimal hyperparameters were identified through a grid search approach, selecting the combination that achieved the highest accuracy in a 3-fold CV. The specific range of hyperparameters used for each ML model in the grid search is presented in Table S1.

## 3. Results and Discussion

### 3.1. Flow Profiles on Paper-Based Microfluidic Chip and Theoretical Curve Fit

**Figure 2:**
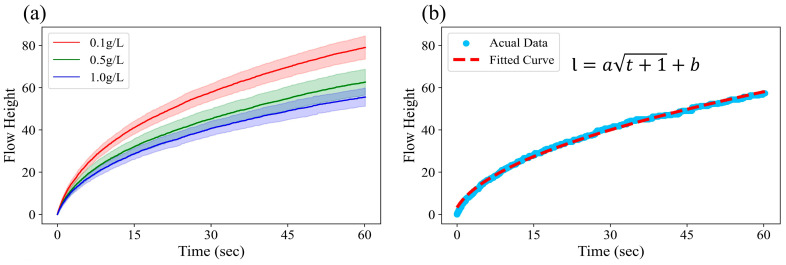

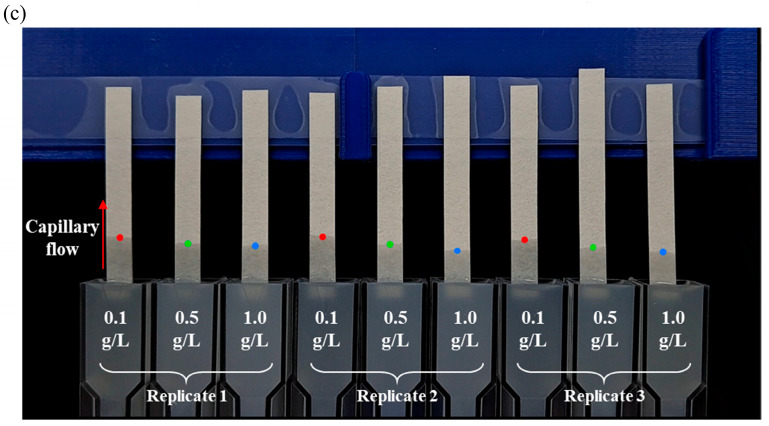
(**a**) Mean flow profiles with the standard deviation for paper-based microfluidic channels using three different tannic acid concentrations: 0.1 g/L (red), 0.5 g/L (green), and 1.0 g/L (blue). The profiles illustrate the capillary-driven advancement of the fluid front over time for 42 samples per concentration, with the shaded areas representing the standard deviation across the replicates. (**b**) An example of a 0.5 g/L flow profile fitted with a theoretical capillary flow model. Dots represent the actual data, while the dashed line indicates the fitted function. The flow height shown on the *y*-axis is defined in Section 2.4.1 and has no units, as it reflects a relative positional difference. (**c**) Snapshots of the capillary flow profiles in nine channels with three different concentrations, demonstrating consistent trends across three independent replicates. Refer to Video S1 to observe the entire flow profile.

Figure 2a shows the flow profiles over 60 s, varying with the concentration of TA. Although there was an overlap in the flow height distributions over time for the 0.5 g/L and 1.0 g/L concentrations, the TA concentration of 0.1 g/L exhibited a significantly faster flow rate compared to the other two concentrations. Intuitive illustrations of this phenomenon are provided in Figure 2c and Video S1.

TA binds to the protein mucin through hydrogen bonding and hydrophobic interactions, forming hydrophobic aggregates and altering the surface tension of the mixture [2,7]. The degree of aggregation between TA and mucin varies with the concentration of TA, leading to changes in the surface tension, which in turn result in differences in the fluid flow profiles on paper-based microfluidic chips.

According to Equation (1), which describes the theoretical flow profile of capillary flow, a higher surface tension results in an increased flow rate. In particular, in paper-based microfluidic chips, the surface tension at the wetting front—the boundary between the wetted and unwetted regions of the paper—plays a critical role in determining the flow rate [13]. The wetting front provides a relatively hydrophobic environment, causing the hydrophobic aggregates formed by the binding of tannic acid and mucin to accumulate more readily at this interface [19]. The accumulation of these hydrophobic aggregates locally reduces the surface tension at the wetting front, thereby weakening the driving force of the capillary flow and decreasing the flow rate [9,13]. In other words, a higher concentration of TA leads to increased formation of hydrophobic aggregates with mucin, resulting in a reduced capillary flow rate.

**Figure 3:**
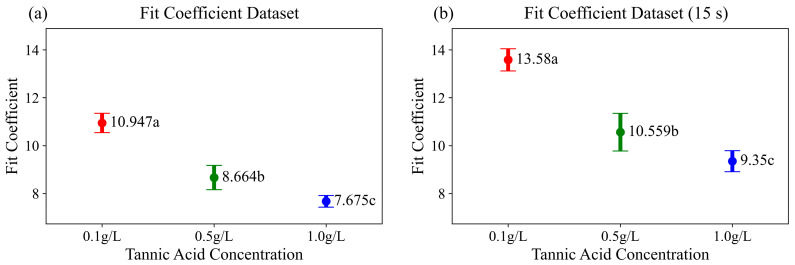
Distribution of the fit coefficient ‘a’ obtained from the theoretical capillary flow model (Equation (3)), corresponding to three different tannic acid concentrations—0.1 g/L (red), 0.5 g/L (green), and 1.0 g/L (blue)—fitted with (**a**) 60 s of data and (**b**) 15 s of data. Different letters indicate significant differences in the mean accuracies based on a one-way ANOVA and Tukey’s HSD test (*p* < 0.05).

As shown in Figure 2b, the TA–mucin mixtures on paper-based microfluidic chips followed the theoretical capillary flow model described by Equation (3). Figure 3a shows that the fitted coefficient *a*, obtained using Equation (3), exhibited a distinguishable distribution across different concentrations of tannic acid. This implies that a robust classification model can be developed using only a single coefficient obtained from the fitted curve, rather than relying on the entire 1800-frame (60 s) profile data, to minimize the computational resources used. Moreover, since the flow behavior closely followed the theoretical profile of the capillary flow function, there is a possibility that the coefficient could be estimated using only a portion of the initial flow data, without requiring the full 60 s dataset. However, when the fitting coefficient was calculated using only the first 15 s of the flow data (Figure 3b), the values were generally higher than those obtained using the entire dataset (Figure 3a). This may be attributed to experimental factors such as variations in the immersion speed or depth, which can cause the actual flow profile to deviate from the theoretical one. Consequently, using a smaller amount of data for fitting may increase the likelihood of deviation from the ideal model profile. Nevertheless, the three groups can still be clearly distinguished using only the initial flow data. Therefore, this suggests that the astringency intensity can be classified in a shorter time and with reduced memory requirements by using the coefficient obtained through curve fitting. A comparison of the ML models’ performance based on this approach is presented in Section 3.2.

### 3.2. Astringency Intensity Classification Using Machine Learning

To identify the most suitable ML model for accurately classifying the TA concentrations based on the flow profiles, four different datasets (raw, mean, fit coefficient, and fit coefficient (15 s)) obtained for the three TA concentrations were used. The 3-fold cross-validation (CV) accuracies of eight ML models were compared, as summarized in Table 1. The hyperparameter combinations selected through 3-fold CV are presented in Table S2.

**Figure 4:**
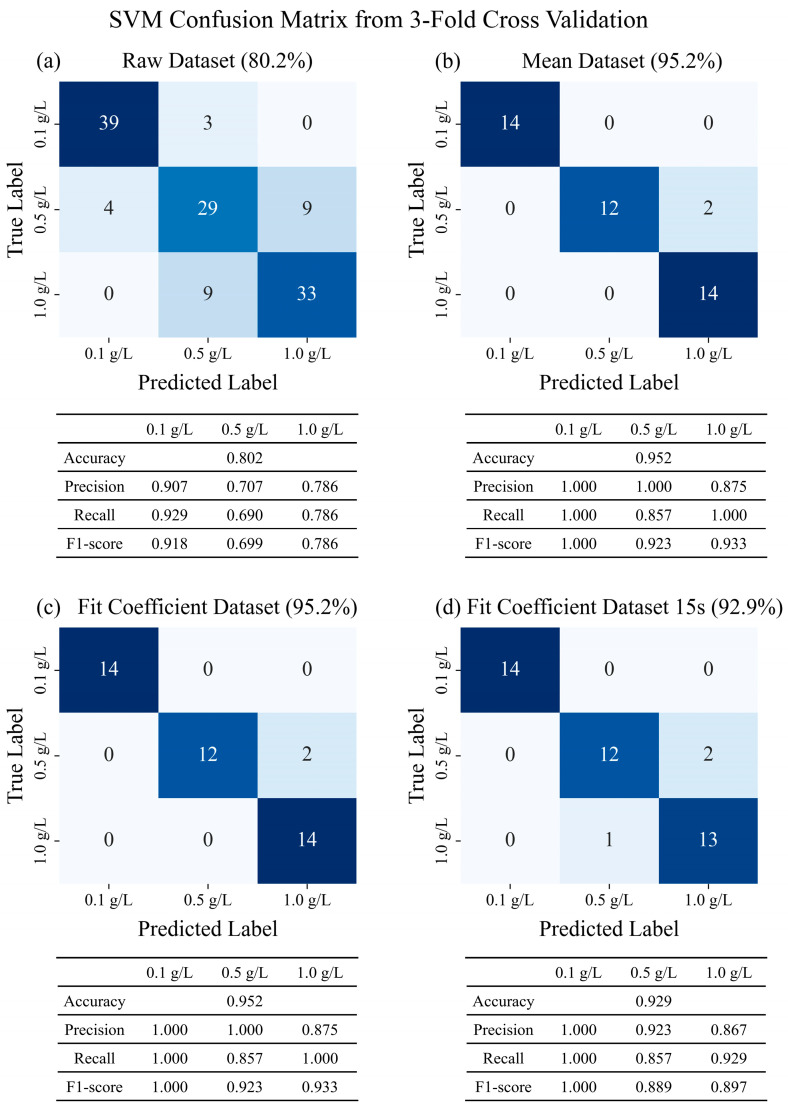
SVM confusion matrix, accuracy, precision, recall, and F1-score from 3-fold cross-validation for tannic acid concentration classification using four types of dataset: (**a**) raw, (**b**) mean, (**c**) fit coefficient, and (**d**) fit coefficient fitted with initial 15 s of profile. Datasets (**a**–**c**) were constructed using full 60 s profiles, whereas dataset (**d**) was derived from only first 15 s of data.

In all the datasets, the SVM model achieved the highest classification accuracy. Therefore, the comparison of the classification performance across the datasets was based on the SVM model. The classification results and performance metrics (precision, recall, and F1-score) for each dataset are presented in Figure 4.

In the raw dataset (row 1), trained using all 126 profiles collected over 60 s, the three groups were classified with an accuracy of 0.80. However, slight variations in the flow, caused by factors such as the cut edges of the paper-based microfluidic chips, non-uniformities in the paper fibers formed during manufacturing, and positional differences during measurement, could have affected the consistency of the flow. Addressing this, a model trained on the mean dataset (row 2), which incorporated the averaged profiles from three independent repetitions for each concentration within a single measurement, achieved a significantly improved accuracy of 0.952.

The model trained using the single coefficient *a* obtained from Equation (3) (row 3) exhibited an accuracy comparable to, or even higher than, that of the model trained on the mean dataset across all the ML models. This is because the coefficient obtained from Equation (3) can be considered an appropriate feature that sufficiently represents the overall flow profile. In addition, since only a single feature was used, the model structure was extremely simple, resulting in minimal differences in accuracy among the eight models. Unlike previous studies [18], which trained models based on the entire capillary flow profile and principal components, this approach can be considered an effective alternative that reduces both the measurement time and model training time.

Furthermore, as shown in Figure 2b, since the capillary flow sufficiently followed the theoretical pattern described by Equation (3), the coefficient could be obtained even when using only a partial set of the initial data (15 s). The model trained based on this approach (row 4) demonstrated performance comparable to that of the model trained using the full 60 s dataset, achieving an accuracy of 0.929. These results are consistent with previous findings [17], which demonstrated that high classification accuracy can be achieved by training ML models using only a few seconds of the initial flow profile data.

The approach we attempted to implement in this study must be specific to the quantification of astringency, clearly distinguishing it from other tastes. Unlike sweetness, saltiness, and sourness, which are perceived through taste receptors, astringency arises from the aggregation of polyphenols, such as TA, with mucin proteins, leading to a stiff and dry sensation in the oral cavity [1]. Because mucin exhibits very weak interactions with taste components other than those responsible for astringency, a previous study [7] confirmed that the detection of astringency using mucin was not affected by the presence of other taste substances.

The lowest concentration of TA, the main component responsible for astringency, in this study was set at 0.1 g/L, which was relatively high compared to the much lower sensing ranges (0.005 g/L) reported for electrochemical sensors [6] and ionic hydrogel sensors [7]. However, considering that the reliability of the best estimate thresholds for detection of tannic acid concentrations with the human tongue is 0.12 g/L [26], the lowest concentration (0.1 g/L) set is sufficient for practical applications. In addition, the method proposed in this study can be implemented as a portable system using only a simple camera and paper [18], eliminating the need for complex sensor fabrication processes or expensive equipment.

Regarding the detection range at higher concentrations, Figure 3 shows that the classification accuracy tended to decrease as the concentration increased. This is because, when sufficient polymerization between TA and mucin occurred, the detection accuracy was reduced. According to previous studies, as the concentration of tannins increases, the particle size of mucin–tannin aggregates also increases [27]. However, when the tannin concentration exceeds 3 g/L, no further increase in the particle size is observed [28]. Therefore, to apply this method at higher concentrations, the sample could be diluted before profile analysis.

In addition, the pH has a significant influence on the interaction between tannic acid and mucin, with the strongest aggregation typically occurring at low pH values of around pH 3 [29,30]. Therefore, adjusting the pH could enable the detection of a wider range of TA concentrations. Furthermore, to facilitate immediate on-site application, future studies could explore the use of divalent metal ions, such as Fe^2+^ and Cu^2+^, which are known to strongly promote binding between TA and mucin [31].

## 4. Conclusions

In this study, the phenomenon of tannic acid aggregating with mucin proteins in the oral cavity was replicated on paper-based microfluidic chips, and the resulting changes in the capillary flow profiles were analyzed using ML models to classify three different levels of astringency. To obtain consistent capillary flow profiles, a motor-driven motion platform and a 3D-printed frame were devised.

Depending on the TA concentration, the number of hydrophobic aggregates formed at the wetting front of the paper-based microfluidic chips varied, leading to local changes in the surface tension and, consequently, differences in the flow profiles. Among eight ML models evaluated for their ability to classify TA concentrations, the SVM model achieved the highest performance with an accuracy of 95.2%. The flow profiles of the TA–mucin mixtures were in line with the theoretical equation for capillary action, allowing for the extraction of a fitting coefficient as a feature. A model trained using this single coefficient achieved performance comparable to that of models trained on full profile data.

Furthermore, by leveraging the fact that the flow profile adhered closely to the theoretical behavior, the fitting coefficient could be extracted using only the initial 15 s of the data. The resulting model achieved a classification accuracy of 92.9%, comparable to that of the model trained with the full 60 s dataset. These results suggest that this approach can be effectively utilized to reduce the measurement, training, and analysis time in future studies involving capillary flow-based profile analyses.

While this study focused on the classification of the astringency intensity based on TA, future work can expand the applicability of this approach to food samples, such as wine or tea, to enhance its practical utility. Structurally, the system can be implemented as a portable device using only a simple camera and paper [18], with uniform sample loading achieved through servomotor control. Based on variations in the interaction capabilities that drive capillary flow, this method offers a low-cost and effective solution for on-site quality assessment and classification of various liquid samples, demonstrating broad applicability across diverse sample types.

## Figures and Tables

**Table 1:** Accuracies and standard deviations from 3-fold cross-validation for eight machine learning models using four dataset types with hyperparameters determined using the grid search method. The raw dataset (row 1) and mean dataset (row 2) were trained using the full 60 s profile data. The fit coefficient dataset (row 3) was trained with a single coefficient derived from the 60 s data and the fit coefficient (15 s) in row 4 with a single coefficient derived from the initial 15 s data.

	Accuracy (Standard Deviation)							
	SVM	LR	MLP	KNNs	RF	LDA	NB	DT
Raw dataset	0.802 (0.030)	0.786 (0.019)	0.770 (0.030)	0.762 (0.039)	0.794 (0.040)	0.778 (0.040)	0.746 (0.040)	0.762 (0.067)
Mean dataset	0.952 (0.067)	0.905 (0.089)	0.929 (0.058)	0.905 (0.067)	0.929 (0.058)	0.952 (0.067)	0.881 (0.089)	0.929 (0.058)
Fit coefficient dataset	0.952 (0.067)	0.929 (0.058)	0.952 (0.067)	0.952 (0.067)	0.952 (0.067)	0.952 (0.067)	0.952 (0.067)	0.952 (0.067)
Fit coefficient dataset (15 s)	0.929 (0.058)	0.881 (0.089)	0.881 (0.089)	0.881 (0.089)	0.881 (0.089)	0.857 (0.058)	0.881 (0.089)	0.857 (0.058)

## Data Availability

Data will be made available on request.

## Supplementary Materials

The following supporting information can be downloaded at: https://www.mdpi.com/article/10.3390/s25165068/s1, Table S1: The hyperparameter combinations for model tuning based on grid search method for each machine learning model. Table S2: The determined hyperparameters of each machine learning model for two different datasets (mean and fit coefficient). Parameters not considered in the grid search were set to their default values. Video S1: Video of motor-based sample loading and flow profile changes in paper chips depending on tannic acid concentration
