# Supplementary material for: Capillary Flow Profile Analysis on Paper-Based Microfluidic Chips for Classifying Astringency Intensity
Source: Sensors (Basel). 2025 Aug 14;25(16):5068. doi: 10.3390/s25165068 (PMC12389808; doi:10.3390/s25165068)
Supplement: Supplementary file 1 [file sensors-25-05068-s001.zip › sensors-3723514-supplementary.pdf]

Supplementary Data

Table S1. The hyperparameter combinations for model tuning based on grid search method for each machine learning model.

ML Model	Hyperparameter combinations
SVM	'kernel': ['linear', 'rbf', 'poly', 'sigmoid'], 'C': [0.1, 1, 10, 20]
LR	'C': [0.1, 1, 10, 100]
MLP	'hidden_layer_sizes': [(3,), (5,), (10,), (3, 3), (5, 3), (10, 3)], 'activation': ['relu', 'tanh']
kNN	'n_neighbors': [3, 5, 7], 'leaf_size': [5, 10, 20], 'p': [1,2,3]
RF	'n_estimators': [10, 20, 30, 40], 'max_depth': [10, 20, 30, 40], 'min_samples_split': [1,2,3,5], 'min_samples_leaf': [1,2,3,5]
LDA	'solver': ['svd','lsqr']
NB	Basic Gaussian Navie Bayes was used
DT	'max_depth': [2, 3, 5], 'min_samples_split': [3,5,7], 'min_samples_leaf': [2,3,5]

Table S2. The determined hyperparameters of each machine learning model for two different datasets (mean and fit coefficient). Parameters not considered in the grid search were set to their default values.

ML Model		Hyperparameter combinations
SVM	Mean Dataset	kernel = ‘linear’, C = ‘1’, class_weight = None
	Fit coefficient Dataset (60s)	kernel = ‘rbf’, C = ‘1’, class_weight = None
LR	Mean Dataset	C=1
	Fit coefficient Dataset (60s)	C=10
MLP	Mean Dataset	hidden_layer_sizes = (10,3), activation = 'relu'
	Fit coefficient Dataset (60s)	hidden_layer_sizes = (3,), activation = 'tanh'
kNN	Mean Dataset	n_neighbors = 3, weights = ‘distance’, leaf_size = 10, p = 2
	Fit coefficient Dataset (60s)	n_neighbors = 3, weights = ‘distance’, leaf_size = 5, p = 1
RF	Mean Dataset	n_estimators = 30, max_depth = 30, min_samples_split = 2, min_samples_leaf = 1
	Fit coefficient Dataset (60s)	n_estimators = 10, max_depth = 10, min_samples_split = 3, min_samples_leaf = 1
LDA	Mean Dataset	solver = 'svd'
	Fit coefficient Dataset (60s)	solver = 'svd'
NB	Mean Dataset	Gaussian naïve bayes model used, var_smoothing = 1e-9
	Fit coefficient Dataset (60s)	Gaussian naïve bayes model used, var_smoothing = 1e-9
DT	Mean Dataset	max_depth = 3, min_samples_split = 3, min_samples_leaf = 3
	Fit coefficient Dataset (60s)	max_depth = 2, min_samples_split = 2, min_samples_leaf = 3
